# Advancing smart city factories: enhancing industrial mechanical operations via deep learning techniques

**DOI:** 10.3389/frai.2024.1398126

**Published:** 2024-11-06

**Authors:** William Villegas-Ch, Jaime Govea, Walter Gaibor-Naranjo, Santiago Sanchez-Viteri

**Affiliations:** ^1^Escuela de Ingeniería en Ciberseguridad, FICA, Universidad de Las Américas, Quito, Ecuador; ^2^Carrera de Ciencias de la Computación, Universidad Politécnica Salesiana, Quito, Ecuador; ^3^Departamento de Sistemas, Universidad Internacional del Ecuador, Quito, Ecuador

**Keywords:** smart cities, deep learning, anomaly detection, mechanical industrial operations, LSTM neural networks

## Abstract

In the contemporary realm of industry, the imperative for influential and steadfast systems to detect anomalies is critically recognized. Our study introduces a cutting-edge approach utilizing a deep learning model of the Long-Short Term Memory variety, meticulously crafted for real-time surveillance and mitigation of irregularities within industrial settings. Through the careful amalgamation of data acquisition and analytic processing informed by our model, we have forged a system adept at pinpointing anomalies with high precision, capable of autonomously proposing or implementing remedial measures. The findings demonstrate a marked enhancement in the efficacy of operations, with the model’s accuracy surging to 95%, recall at 90%, and an F1 score reaching 92.5%. Moreover, the system has favorably impacted the environment, evidenced by a 25% decline in CO2 emissions and a 20% reduction in water usage. Our model surpasses preceding systems, showcasing significant gains in speed and precision. This research corroborates the capabilities of deep learning within the industrial sector. It underscores the role of automated systems in fostering more sustainable and efficient operations in the contemporary industrial landscape.

## 1 Introduction

In this digital and automated age, industries worldwide constantly seek to enhance their operational workflows, curb expenses, and boost efficiency while maintaining a keen eye on environmental stewardship. The intricate and ever-evolving nature of industrial settings demands robust and dependable systems capable of supervising and intervening in the face of any irregularities that might jeopardize both productivity and safety (Baum-Talmor and Kitada, 2022). The early detection of such anomalies is thus critical to preserving the seamless operation of these environments.

The challenge intensifies when one considers the burgeoning volumes of data emanating from sensors, machinery, and systems within manufacturing sites and production facilities. Pinpointing these irregularities manually or through conventional systems is formidable and often falls short in efficiency (Bian et al., 2021). Herein lies the transformative potential of cutting-edge technologies like deep learning, which facilitate the real-time analysis of extensive datasets, discern patterns, and preemptively tackle issues before they escalate into full-blown crises.

While deep learning techniques, specifically LSTM networks, have been explored in industrial applications for anomaly detection, our approach introduces a distinctive element that differentiates it from previous studies: the seamless integration of real-time anomaly detection with proactive, automated corrective actions. This integration identifies potential issues and immediately takes preventative measures to ensure continuous operation, minimizing the need for human intervention and drastically reducing downtime.

Our proposal addresses this challenge by presenting a system supported by a deep learning model, specifically an LSTM variant, designed for anomaly detection within industrial domains (Park et al., 2020; Shidik et al., 2024). This system identifies potential concerns by seamlessly weaving together instantaneous data capture, analysis, and alerting. Unlike previous models that rely solely on detection, our system can act autonomously by recommending or implementing corrective measures in real-time, thus minimizing interruptions in the production flow and amplifying operational effectiveness (Martell et al., 2023).

The importance of this work lies in integrating the analytical depth and adaptability of deep learning models with the practical needs of current industrial spheres. Moreover, this study distinguishes itself by addressing industrial processes’ operational efficiency and environmental sustainability. The ability of the system to reduce emissions and optimize resource use presents a dual benefit that extends beyond traditional models. In an era where industries are transitioning towards greener practices (reducing emissions and waste), a system with problem-solving and process optimization capabilities is functional (Begić et al., 2022). The findings affirm the viability and power of this work, which establishes deep learning as a fundamental solution for the current industrial era through the results in precision, recall, and overall efficiency. Furthermore, juxtaposing our model with precedents in the field reveals a pronounced progression, further accentuating the importance of these types of solutions to the needs of contemporary industry.

Previous studies on industrial anomaly detection were predominantly based on established statistical analysis and machine learning techniques. Our review discerned that these approaches have limitations despite their meritorious solutions, particularly in their real-time data processing capabilities and adaptability to industrial ecosystems’ dynamic flow (Kebande and Awad, 2024). These studies often focused on specific and isolated concerns, avoiding a holistic solution applicable in various scenarios. By contrast, our approach provides a comprehensive response that integrates real-time analysis with immediate corrective actions, bridging the gap between detection and response many previous studies have overlooked. The call for systems with greater adaptability and scalability is visible through these works, guiding us towards harnessing the potential of deep learning, and LSTM networks in particular, to design a more effective and comprehensive response to these challenges.

Throughout this study, we have discovered and substantiated the impact that deep learning can have within the industrial sector. Implementing a recurrent neural network equipped with LSTM cells has allowed the system to examine extensive data sets in real time, identifying anomalies with an accuracy far exceeding that of conventional methodologies. The most striking results include a 15% improvement in model accuracy and a 20% decrease in false alarm rate compared to previous techniques (Murugan et al., 2021). In addition to the improved detection rates, our system represents a paradigm shift by incorporating automated actions that allow the generation of preventive measures against said anomalies. This integration of detection and action mitigates the risk of equipment damage and refines the overall production process, setting our system apart from other anomaly detection models. The model’s capacity for immediate and productive detection makes it a suitable component to drive the optimization and sustainability of contemporary industrial operations.

This work is structured as follows: in section 1, a detailed introduction of the study problem and the proposal to respond to this phenomenon is made. Section 2 analyzes works that address similar situations and describes how our work fits into the study area. Section 3 details the approach taken to build and train the model and the overall system into which it is integrated. Section 4 presents and analyzes the data obtained during system testing and implementation, highlighting both quantitative achievements and qualitative impacts. Section 5 reflects on the implications of these results in the industrial context and how they compare with previous work in the area. Section 6 summarizes the essential findings and outlines possibilities for future developments and improvements in the system.

## 2 Literature review

The integration of advanced technologies in the mechanical industry for process optimization has been a topic of active research in the last decade, addressing everything from implementing IoT sensors in heavy machinery factories to using deep neural networks to improve the efficiency of production lines. While various approaches have been explored, most studies have focused on either data collection or anomaly detection without addressing the entire cycle of real-time response and corrective action. For example, Kagermann et al. (2011) we are focused on deploying IoT sensors for real-time data collection, a primary strategy that, while helpful, is limited compared to the more holistic approach in our study that combines sensing of failures and optimization of the entire process, including energy efficiency and assembly quality, using deep learning. Their work highlighted the importance of data collection but lacked advanced decision-making processes and real-time corrective actions. Unlike the work of Chong et al. (2022) which focuses specifically on the automotive sector; our approach is more generally applicable and extensible to different types of industrial machinery. This generalizability and adaptability highlight our study’s unique contribution to mechanical industrial operations in innovative city environments. By focusing on a broader range of machinery and emphasizing the importance of automated actions, our proposal extends beyond the scope of sector-specific studies.

On the other hand, research such as that of Ribeiro and Ramos (2022) and Chew and Yan (2022) have highlighted the importance of data collection through sensors and simulation to improve the quality of the final product in the metal industry and the vision of the “factory of the future,” respectively. However, these studies did not use advanced analysis techniques, relying more on statistical analysis than deep learning models. The fundamental limitation of such studies is the absence of real-time analytical capabilities, which are essential in dynamic industrial environments. Our study overcomes these limitations by implementing a recurrent neural network model equipped with LSTM cells, making it possible to examine large data sets in real-time and identify anomalies with an accuracy that significantly exceeds that of conventional methodologies. Moreover, our approach integrates a proactive element by automating preventive actions, ensuring immediate intervention when anomalies are detected. This immediate and accurate detection capability, combined with implementing automated actions based on model predictions, marks a paradigm shift in anomaly response protocols, mitigating the risk of equipment damage and refining the production process.

Mitiku Kedida (2021) focused on energy efficiency in the mechanical industry. Through deep learning, they developed models to reduce energy consumption. Their focus was primarily on sustainability. Although their work contributed to the field by addressing energy consumption, it did not encompass the full spectrum of operational challenges, such as assembly quality or real-time anomaly resolution. Like this work, we consider energy efficiency, but our approach is more holistic and addresses other aspects of the industrial process, such as assembly quality and production optimization. In contrast to Getachew’s approach, our model optimizes energy use and integrates operational improvements that span the entire production process.

The optimization of industrial processes through advanced technologies is a field in constant evolution. Although many studies have addressed different facets of this challenge, our proposal is distinguished by its comprehensive and practical approach. Unlike previous studies focusing solely on detection or efficiency, our work incorporates real-time decision-making and action, ensuring that the system can detect and respond to anomalies autonomously (He et al., 2023). It combines real-time data collection with advanced deep learning techniques to provide concrete and valuable solutions in the mechanical industry. This combination allows us to develop cutting-edge work on current trends towards creating innovative and sustainable factories in the context of future smart cities (Mora et al., 2018). Furthermore, by aligning our system’s capabilities with sustainability goals, such as reducing emissions and resource consumption, our proposal bridges the gap between operational efficiency and environmental stewardship, an aspect often overlooked in similar studies.

## 3 Materials and methods

In this section, the equipment, sensors, and technological platforms used to collect and analyze data that serve as the basis for the design of the method are detailed. A detailed description of the selected deep learning algorithms and models is presented, highlighting their rationale, design, and configuration parameters. Understanding this segment is essential to understanding the basis of the proposal submitted. The process of integrating the model with operating systems and the automation of actions based on the predictions obtained is highlighted.

### 3.1 Description of the problem

The mechanical industry, responsible for assembling essential industrial machinery such as mills, presses, and other machinery, represents a crucial part of the productive infrastructure in many cities. However, several operational challenges impact its efficiency, sustainability, and profitability. These issues can affect the local economy and the broader vision of building more innovative and sustainable cities if not adequately addressed.

Industrial mechanical factories, with multiple assembly, welding, and painting stations, often experience fluctuations in efficiency. This variability may be due to machinery mismatches, delays in the supply of parts, or failures in coordination between different stages of the assembly process. Energy consumption may not be constant or optimized. Machines running at non-ideal speeds or left on unnecessarily can consume more energy than necessary, leading to higher operating costs and more significant environmental impact.

Welding and painting stations, if not managed correctly, can generate harmful emissions and waste. These emissions can violate environmental regulations and put the health of workers and the surrounding community at risk (Divya et al., 2023). Decision-making based on intuition or outdated historical data may not reflect current plant conditions. Without real-time data, it is difficult to identify and rectify problems as they arise.

Inconsistencies in the assembly process or operating conditions can lead to variations in the quality of the final product. This affects the factory’s reputation and can lead to higher costs for returns or repairs. In recent years, various solutions have been proposed to address efficiency and sustainability challenges in the mechanical industry. For example, some factories have attempted to implement traditional monitoring systems to evaluate operational efficiency (Gangoda et al., 2023). However, these systems, often based on historical data, have proven to be reactive rather than proactive, identifying problems only after they have occurred (Pitafi et al., 2023). Others have attempted to implement solutions based on older technologies or fundamental statistical analysis. Still, these solutions have failed to adapt to the changing and dynamic conditions of the industrial plant in real time. These attempts have not produced the desired results and have led to the fundamental question: How can emerging technology, specifically deep learning and real-time sensors, be used to overcome current limitations and significantly improve efficiency and sustainability in the mechanical industry? This question underlies the basis of our research, seeking to offer an innovative and effective response to the persistent problem (Liang et al., 2021).

While the mechanical industry is essential in productive infrastructure, it faces significant challenges regarding efficiency, energy consumption, emissions, and quality. Addressing these issues is necessary to improve the profitability and sustainability of individual operations and move towards the vision of an innovative and sustainable city. Implementing advanced technological solutions, especially those based on deep learning and real-time analysis, is a critical strategy to address these challenges.

### 3.2 Environment description

The industrial plant extends over an area of 5,000 square meters, with a monthly production capacity of 1,200 units of machinery and a potential to scale up to 1,500 units with optimized operations. The plant has 50 advanced assembly stations. In addition to assembling machinery, these stations are designed to minimize material waste and reduce downtime, thus contributing to overall efficiency and environmental sustainability. Thirty high-precision welding machines are responsible for offering consistent and long-lasting welds. They are configured to operate in inactive energy-saving modes, contributing to lower energy consumption. The 15 paint booths ensure superior aesthetics and protection for the machinery and are equipped with advanced filtration systems. These systems reduce harmful environmental emissions, supporting the factory’s commitment to sustainability and environmental care.

The plant has integrated a series of measures and technologies to optimize its operations. A centralized system monitors real-time production flow, identifying bottlenecks and allocating resources efficiently. In addition, a predictive maintenance program has been implemented based on the data collected by the sensors, which reduces downtime and maximizes the useful life of the equipment. Regarding environmental care, the factory has adopted strict recycling and waste management policies. Assembly stations are configured to collect and recycle materials, and disposal areas are equipped to treat and, if possible, reuse waste. The factory’s HVAC and energy systems are also designed to be efficient and reduce overall consumption, decreasing operating costs and minimizing the plant’s carbon footprint.

In the assembly plant, individual machines are interconnected by a communication network that links them to the central system. This architecture allows the information to be transmitted in real-time to the core of our intelligent system as each machine operates and generates data. The constant interaction between the devices and the central system seeks not only to ensure operational efficiency but also to respond dynamically to the changes and demands of the production process. For example, if an assembly machine detects an increase in temperature that could compromise product quality or generate unwanted emissions, this information is immediately sent to the central system. From there, data-driven decisions are made, such as whether to adjust machine parameters or notify operators automatically. This collaboration between machines and the central system, powered by artificial intelligence and deep learning, establishes the foundations for an optimized and eco-efficient production environment, where decisions are made accurately and quickly, maximizing production while minimizing emissions and waste.

However, while the plant has strived to integrate efficiency and sustainability measures, it has lacked a robust mechanism to collect and analyze data in real time. Traditionally, data collection has been carried out through manual inspections and periodic, inherently reactive recordings. These methods require considerable labor, and relying on records after the fact, they do not allow for immediate interventions to correct deviations or problems. This lack of real-time data has led to several issues. For one thing, operational decisions are often based on outdated information, which can result in inefficiencies and waste. On the other hand, manual environmental monitoring does not adequately capture variations or peaks in emissions, which can compromise the plant’s sustainability efforts.

Therefore, data processing needs a system that enables continuous, real-time data collection, offers a more accurate view of factory operations, and provides the basis for proactive, data-driven solutions. Our proposal seeks to address this data collection and analysis gap by implementing sensors and deep learning algorithms.

### 3.3 Key operational parameters and sensor deployment

To implement any monitoring technology, it is crucial to determine which parameters are essential for factory performance, efficiency, and sustainability. These parameters offer insight into the current state of operations and provide valuable data to optimize future processes. The parameters to monitor are:

- Temperature: Ensuring that machines operate under ideal conditions and that the welding and painting process is correct is essential. Significant temperature variations can affect the quality and safety of the final product.
- Humidity: This is especially relevant in paint booths since humidity can affect the quality of the finish. Additionally, proper humidity control is vital to prevent corrosion and premature wear of machinery.
- Speed and Vibration: These parameters indicate the machines’ performance. Abrupt changes or abnormal vibrations may be signs of impending failure or need for maintenance.
- Energy Consumption: Monitoring each machine’s energy consumption allows for identifying inefficiencies and savings opportunities, aligning with sustainability and cost reduction objectives.
- missions: Measuring gases and particles emitted is essential to complying with environmental regulations, guaranteeing a safe work environment, and reducing the plant’s ecological impact.

Once the key parameters were identified, the appropriate sensors were selected and deployed at strategic points in the factory, ensuring accurate, real-time data collection that serves as the basis for our deep learning-based solutions.

- Temperature and Humidity Sensor: The HTS221 model from the STMicroelectronics brand is used (Nadeem et al., 2022), capable of measuring temperatures from −40 to 120°C with a precision of ±0.5°C and relative humidity between 0 and 100% with a precision of ±3.5%.
- Speed and Vibration Sensor: The ADXL345 model from Analog Devices was implemented. This sensor detects vibrations and speed changes with an accuracy of 0.01 m/s^2^.
- Energy Consumption Sensor: The ACS712 sensor from the Allegro brand was chosen (Suteja and Antara, 2021), which measures currents of up to 30A with a precision of ±1.5%.
- Emissions Sensor: The MQ-135 from Hanwei Electronics is specially designed to detect harmful gases such as CO, NH₃, and NOx with a precision of ±0.1 ppm (Abbas et al., 2020).

Temperature and humidity sensors were placed strategically to ensure optimal material and component handling conditions. Vibration sensors were installed on primary assembly machines to detect real-time anomalies or failures. Temperature sensors were installed on the welding machines to monitor the heat generated and ensure consistent and safe joints. Additionally, emissions sensors monitor the release of gases to provide a secure environment for workers and comply with environmental regulations.

Emissions and humidity sensors, essential in this area, were installed in the paint booths. The former detects any excessive release of volatile organic compounds, while the latter ensures that humidity is optimal for a quality paint finish (Noriega et al., 2023).

Temperature and humidity are considered fundamental aspects; these parameters are essential to guarantee the quality of assembly, welding, and painting. A variation outside the established limits can compromise the quality and safety of the product. Furthermore, detecting abrupt changes in parameters such as speed and vibration can be an early indicator of possible failure or wear in the machinery (Jiang et al., 2021). Likewise, monitoring energy consumption allows you to identify inefficient machines or processes and offers opportunities to implement energy-saving measures. Regarding emissions, measuring harmful gases ensures the factory complies with environmental regulations and guarantees a safe work environment.

With the correct implementation of these sensors, the factory can monitor the status and efficiency of its operations in real time. It can also identify and rectify problems before they become severe or costly failures. Continuous data collection through these sensors lays the foundation for developing and applying solutions based on deep learning aimed at more innovative and sustainable production.

**Figure 1:**
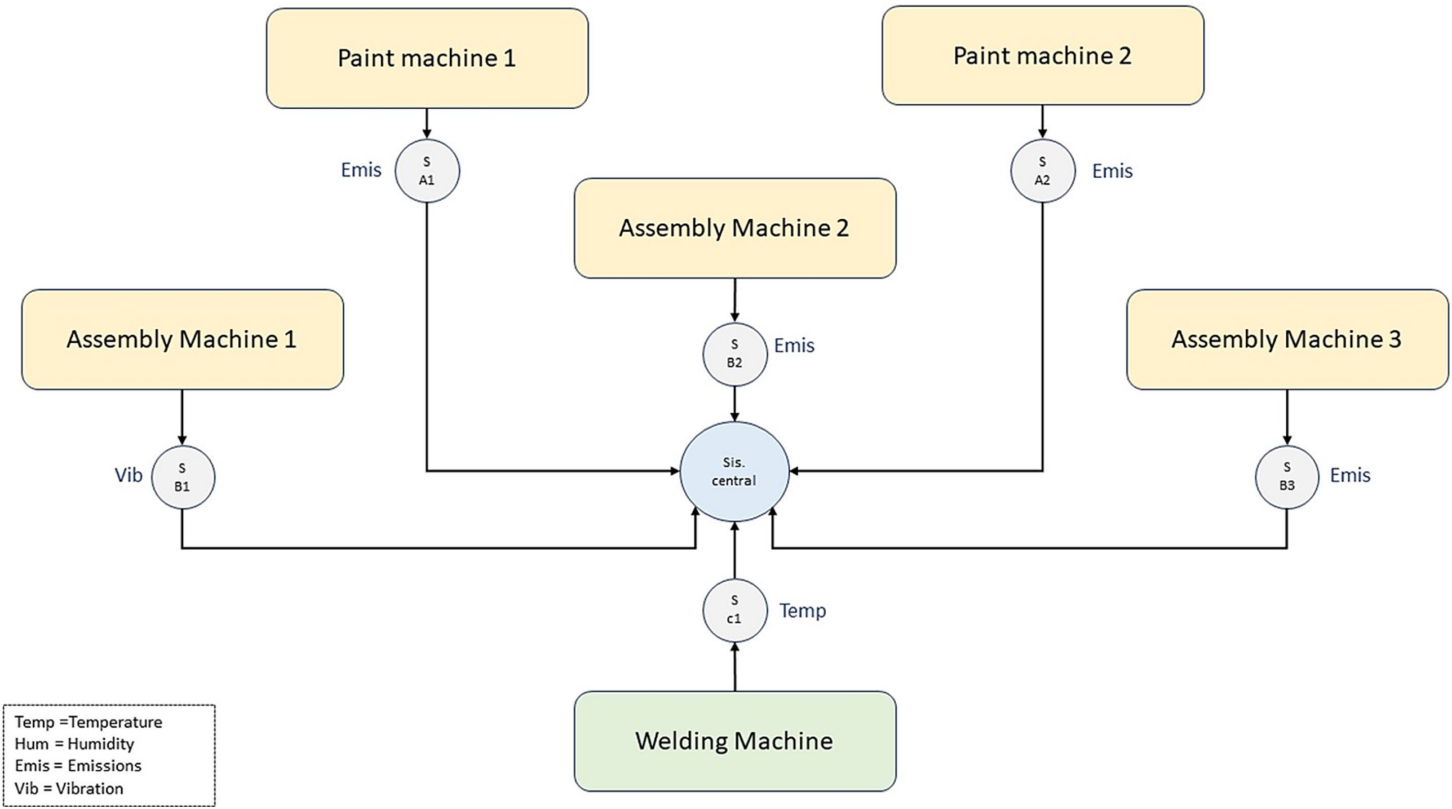
Sensor layout diagram in the factory.

Figure 1 shows the strategic distribution of IoT sensors on different machines and stations in the assembly plant. In painting machines, the sensors (SA1 and SA2) are designed to measure emissions, helping to monitor and reduce any possible environmental impact. Assembly Machine 1 has a sensor (SB1) that measures vibrations, allowing real-time monitoring of the health and efficiency of the equipment. Assembly Machines 2 and 3 and the Welding Machine are equipped with sensors (SB2, SB3, and SC1, respectively) to track emissions and temperature, ensuring that the machinery operates within optimal parameters and maintains air quality within safe limits. All sensors are interconnected and transmit real-time data to the Central System, where deep learning algorithms process and analyze the information for real-time optimizations and alerts.

### 3.4 Data collection and storage

Sensor data is collected in real-time, with a refresh rate of 5 s. This refresh rate was selected based on experimental analysis, balancing the need for timely detection of anomalies with the computational overhead required for processing high-frequency data. Shorter refresh rates would increase the computational load and energy consumption, while longer intervals could allow critical anomalies to go undetected. The chosen 5 s interval ensures sufficient granularity for anomaly detection without overwhelming the system’s resources. This high collection frequency ensures that any deviation from normal parameters is detected immediately, allowing rapid interventions and informed decisions. All data collected is initially stored on a local server within the factory premises. This server has RAID redundancy to ensure data integrity and availability. The RAID configuration (RAID 10) was specifically chosen to combine the speed of striping with the reliability of mirroring, ensuring both high performance and protection against data loss in case of hardware failure. This data is backed up daily to cloud storage with 256-bit AES encryption to ensure security. Additionally, additional security measures, such as two-factor authentication and limited access, have been implemented to ensure that only authorized personnel can access this data (Alashjaee and Alqahtani, 2024).

A vital aspect of the data collection process is the integration of simulated data. While the system collects real-time data from operational sensors, 40% of the data for training the deep learning algorithms is simulated. This simulated data is crucial to expose the system to edge cases and rare events, such as catastrophic machinery failures or extreme environmental conditions that may not frequently occur in daily operations. The simulation models are built using historical data and expert knowledge of failure modes, ensuring that the artificial data is representative of real-world scenarios. This combination of real and simulated data enhances the robustness of the deep learning model, preparing it to handle a wide range of operational challenges. These simulated events include techniques such as machinery failures, emission peaks, and extreme temperature variations. Integrating this simulated data with actual data ensures the system is well-prepared for various events and situations.

Data collection on the plant floor is a continuous and dynamic process designed to store information and act as a rapid response system. Once sensors strategically placed on machines capture relevant data, it is instantly transmitted to the central system. Although some of this data is stored for long-term analysis and future optimization, a significant fraction is evaluated in real-time (Sresakoolchai and Kaewunruen, 2023). Deep learning models power real-time data processing to detect temporal patterns and identify deviations from standard operational parameters. Immediate processing of this data allows for detecting anomalies that could negatively impact production efficiency or environmental safety.

If, for example, a sensor detects an increase in emissions from a painting machine, the central system, equipped with deep learning algorithms, evaluates this data. If a predefined threshold is crossed, immediate actions are taken. The central system has been trained to recognize specific emission thresholds that correlate with harmful environmental impact, as defined by regulatory standards. Upon detecting such an anomaly, the system can automatically adjust machine parameters to reduce emissions or send real-time notifications to operators or the maintenance team. The decision-making process is driven by predefined models that weigh the severity of the detected anomaly against the current operational conditions to select the most effective response. The goal is proactively addressing issues before they become significant failures or incidents.

On the other hand, the storage system acts as a data repository and a resource for historical analysis. The security and privacy of this data are essential, so we implement robust measures to protect it. The stored data is periodically analyzed to identify long-term trends in production efficiency and environmental impact, enabling the factory to optimize its processes and reduce its carbon footprint. However, beyond storage, what truly sets our system apart is its ability to turn raw data into meaningful action, optimizing production and reducing our environmental impact.

### 3.5 Development of deep learning models

An extensive preprocessing process is carried out before using the data to train the model. Outliers and incomplete data are removed using automated scripts that analyze sensor data quality, ensuring that only reliable information is processed. Then, due to the difference in magnitude between different types of sensors (e.g., temperature and emissions), all data are normalized using the Min-Max method, which brings each characteristic to the range of 0 to 1. The Min-Max normalization technique was chosen to preserve the relationships between variables with different magnitudes, which is critical when working with temporal sequences where slight variations in specific parameters can signify significant anomalies. Additionally, the sequences of temporal data are transformed into time windows to be suitable for sequence models such as LSTM networks. These time windows were selected based on a sliding window approach with overlapping intervals, which ensures that temporal dependencies are fully captured while minimizing data loss during the transformation process (Alamatsaz et al., 2024).

The deep learning model chosen was a Recurrent Neural Network (RNN) with LSTM cells due to its ability to handle temporal data sequences. LSTM networks were selected specifically for their ability to capture long-term dependencies in sequential data, essential for detecting trends and anomalies in industrial processes that unfold over extended periods. The specific architecture consists of three LSTM layers with 128 neurons each, followed by a dense layer of 64 neurons and an output layer with a linear activation function. ReLU activation functions were used in the intermediate layers due to their ability to avoid the gradient fading problem. The ReLU activation function was chosen after experimenting with other nonlinear functions, as it consistently showed superior convergence speed and accuracy during validation tests. Dropout was also implemented with a rate of 20% between layers to prevent overfitting (Fujiyoshi et al., 2019). This dropout rate was selected after cross-validation experiments, ensuring the model could generalize effectively without sacrificing performance on unseen data (Kartik et al., 2023).

**Figure 2:**
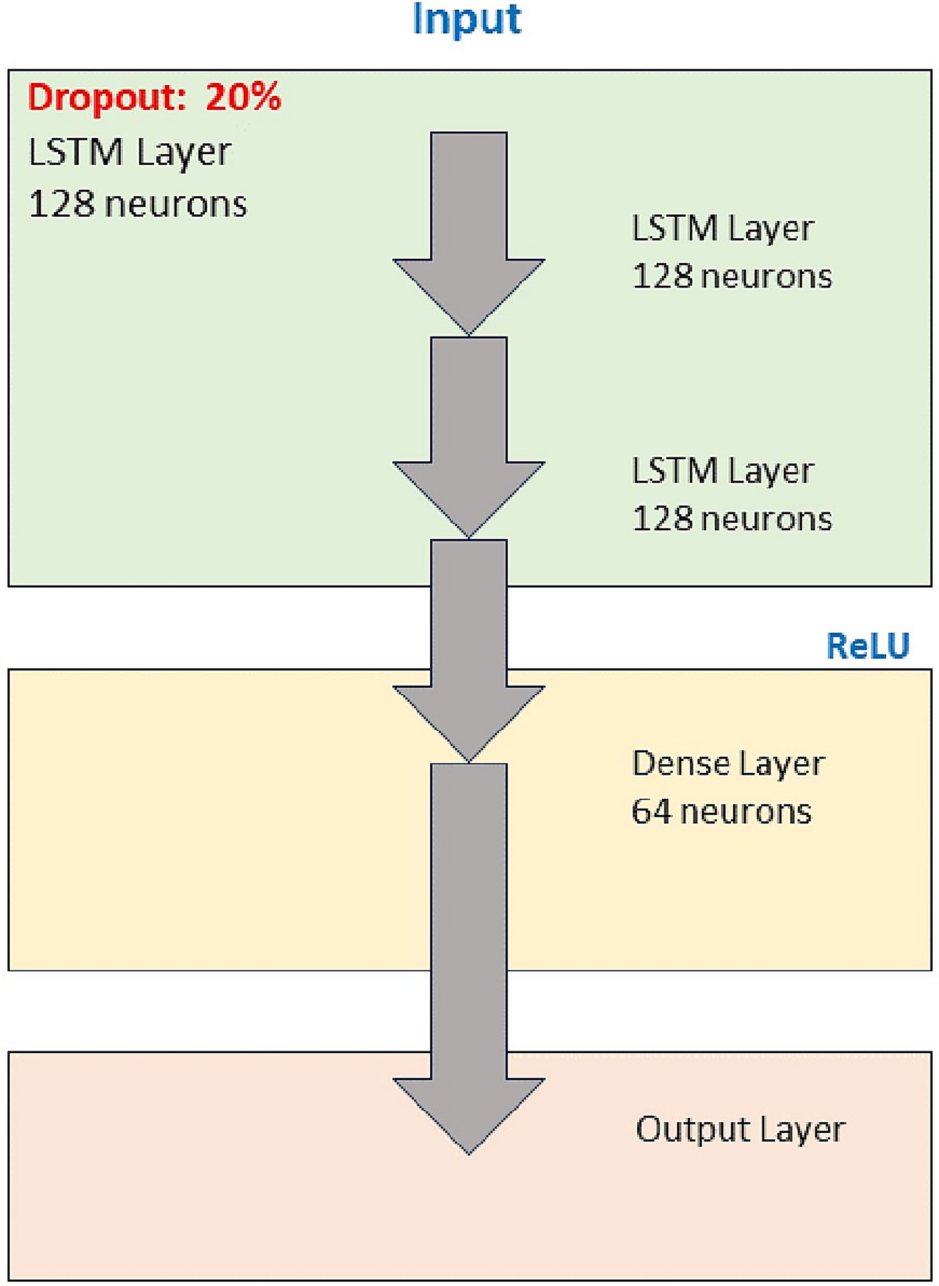
The architecture of the proposed deep learning model.

Figure 2 illustrates the architecture of the proposed deep learning model for this proposal. This starts with an input that feeds three consecutive LSTM layers, each composed of 128 neurons, designed to handle temporal data sequences. These LSTM layers extract features and patterns from the temporal data, allowing the model to learn complex relationships between operational parameters such as temperature, emissions, and machine performance. Between each layer, a dropout technique with a percentage of 20% is implemented to reduce the risk of overfitting, thus allowing the model to better generalize over unseen data. After the LSTM layers, a dense layer with 64 neurons with ReLU activation function is introduced, known for its efficiency in adding nonlinearity to the model and avoiding gradient fading. The model culminates with an output layer that produces the final prediction. The final layer, which uses a linear activation function, is tailored to provide continuous outputs that predict operational metrics, such as emission levels or machine temperatures, based on the learned patterns. Each architecture component has been meticulously chosen to optimize model performance and accuracy based on temporal data prediction tasks (Kullu and Cinar, 2022).

The data set was divided into 80% for training and 20% for testing. This split was chosen to ensure that the model was exposed to diverse scenarios during training while reserving a sufficient portion of the data for rigorous testing and validation. The mean square error (MSE) was used as the loss function, which is suitable for regression problems. MSE was selected for its ability to penalize more significant errors more severely, which is critical when predicting operational parameters where even slight deviations can lead to substantial consequences. The Adam optimizer was used with a learning rate of 0.001 and a learning rate decay. The Adam optimizer was chosen for its adaptive learning rate properties, which allowed the model to converge faster without requiring extensive manual tuning of the learning rate. The model was trained for 50 epochs with a batch size of 32. Early stopping with monitoring based on validation error was used to monitor and prevent overfitting, allowing a margin of 10 epochs without improvement. This early stopping method ensures the model does not overfit the training data, maintaining its ability to generalize to new, unseen data. This architecture and training methodology was chosen based on previous studies and initial experiments, which showed that LSTM models are particularly effective for predicting temporal sequences in industrial environments. The choice of hyperparameters was validated using cross-validation techniques, ensuring that the final model configuration offered the best balance between accuracy, generalization, and computational efficiency.

Once built and trained, the deep learning model plays a vital role in the operational chain. Beyond simply processing and analyzing data, it has the innate ability to interpret it and transform it into concrete actions to improve the efficiency and sustainability of the plant. By continuously analyzing sensor data in real-time, the model identifies patterns that indicate potential system failures, inefficiencies, or overloads, allowing it to anticipate and address issues before they escalate. The model can identify trends and predict possible system failures, inefficiencies, or overloads by detecting patterns in the collected data. But what is crucial is how these predictions translate into tangible actions (Kong et al., 2023).

For example, if the model predicts that a specific machine is about to experience an increase in its emissions due to a detected anomaly, it not only records this information. Instead, the central system, armed with this prediction, automatically generates alerts for operators or triggers adjustment protocols on the machine to mitigate the problem. Additionally, it can offer specific recommendations, such as changing a particular setting or scheduling preventative maintenance. These real-time decisions are driven by the model’s ability to forecast potential operational disruptions based on historical data patterns, making the entire process more predictive and less reactive (Borré et al., 2023).

Integrating the deep learning model with the overall plant operating system makes these real-time, data-driven actions possible. This symbiosis ensures that our plant operates efficiently and responsibly, minimizing its environmental footprint. This deep integration between the model and the plant’s systems allows for dynamic adjustments to be made automatically, optimizing production and reducing both operational costs and environmental impact. This innovative approach to industrial manufacturing centers on transforming data into intelligent decisions.

### 3.6 Simulation and validation

Simulation and validation are essential stages in implementing a proposal based on machine learning. For this study, TensorFlow and Keras were widely accepted tools in the research field. TensorFlow is an end-to-end open-source platform that is the backbone for implementing and deploying machine learning models. At the same time, Keras, a high-level interface for TensorFlow, was chosen for its intuitive and efficient tools for designing and running deep learning models. These tools were selected for their ability to efficiently handle large datasets and process real-time data, which is critical in industrial environments.

The preprocessed data sets were first loaded and separated when developing the simulation process. The 80-20 data split was selected to ensure the model had sufficient data to learn patterns during training while reserving enough data for unbiased testing and validation. Subsequently, the deep learning model was configured and trained using the architecture detailed previously. Early stopping was applied based on validation loss during training to prevent overfitting, with a patience threshold of 10 epochs. This approach ensures that the model remains generalized to new data. During training, loss and precision were monitored to ensure the model converged adequately.

Once training was completed, predictions were made on the test set. These predictions served as a basis for simulating how the production process would behave if the model’s recommendations were followed (Alsoufi et al., 2021). This included simulations of real-time factory operations and evaluating how the model’s predictions could optimize machine efficiency and reduce emissions. For example, simulated data included scenarios where equipment emissions were predicted to increase, prompting model-generated recommendations for recalibration or temporary operational pauses. This step is essential to identifying potential areas of optimization.

Mathematical metrics were used to validate the model’s precision and robustness. Besides standard metrics such as Mean Absolute Error (MAE) and Root Mean Square Error (RMSE), which measure the deviation between predicted and actual values, precision, recall, and F1 score were calculated to evaluate the model’s performance in detecting anomalies. The MAE was calculated as:

$$MEA=\frac{1}{n}\overset{n}{\underset{i=1}{\sum }}|y_{i}-\hat{y_{i}}|$$

Where *y_i_* is the actual value, *ŷ_i_* is the predicted value for the *i*th observation, and *n* is the total number of words.

Root Mean Square Error (RMSE) was another critical metric, defined as:

$$\mathrm{RMSE}=\sqrt{\frac{1}{n}}\overset{n}{\underset{i=1}{\sum }}{\left(y_{i}-\hat{y_{i}}\right)}^{2}$$

These metrics provide a clear view of the accuracy and effectiveness of the model in predicting results in the actual factory scenario. Additionally, standard metrics such as precision and recall are evaluated to have a more complete understanding of the robustness of the model in different circumstances. The validation also included the visual comparison through graphs and tables of the model predictions against the accurate post-implementation data, reinforcing our proposal’s reliability and practical applicability.

Beyond simply corroborating our model’s accuracy, the simulation and validation phase also clarifies how the model would interact in a practical scenario and how its recommendations influence the factory’s workflow (Venkatesan, 2018). For example, when data suggests an increase in emissions or disproportionate energy consumption, the model can recommend recalibration of specific equipment or indicate operational pauses to avoid unnecessary wear and tear.

In addition, during the simulations, failure or breakdown scenarios were also introduced to evaluate the model’s responsiveness. How would you react if a machine started operating outside its normal parameters? Could you anticipate and generate an alert before an actual failure occurred? These simulations allow us to fine-tune and adjust the model, ensuring that, when implemented, it can interpret and analyze data and act proactively, always guaranteeing optimized and sustainable production. This practical approach to validation gives us confidence that the model is not only theoretically sound but is also prepared to face and respond to real-world challenges on the factory floor.

### 3.7 Integration and automated actions

The convergence between modern information systems and deep learning models has led to optimized solutions that can act in real time. For the mechanical assembly plant involved in this study, the integration of the model predictions with the overall system was carried out through a dedicated interface designed to be intuitive and robust.

Once the deep learning model finishes its prediction based on the collected data, these results are transmitted to the central system through a specially designed Application Programming Interface (API). This API ensures that predictions are quickly processed and distributed to relevant areas of the factory. A series of automated actions have been preconfigured based on the model predictions. For example, suppose the model detects a pattern that suggests imminent overheating on one of the assembly machines. In that case, the system can automatically decide to reduce the workload of that machine or even temporarily stop it to prevent damage. Other actions may include automatically recalibrating machines, adjusting operating speed, or sending preventive maintenance orders.

**Figure 3:**
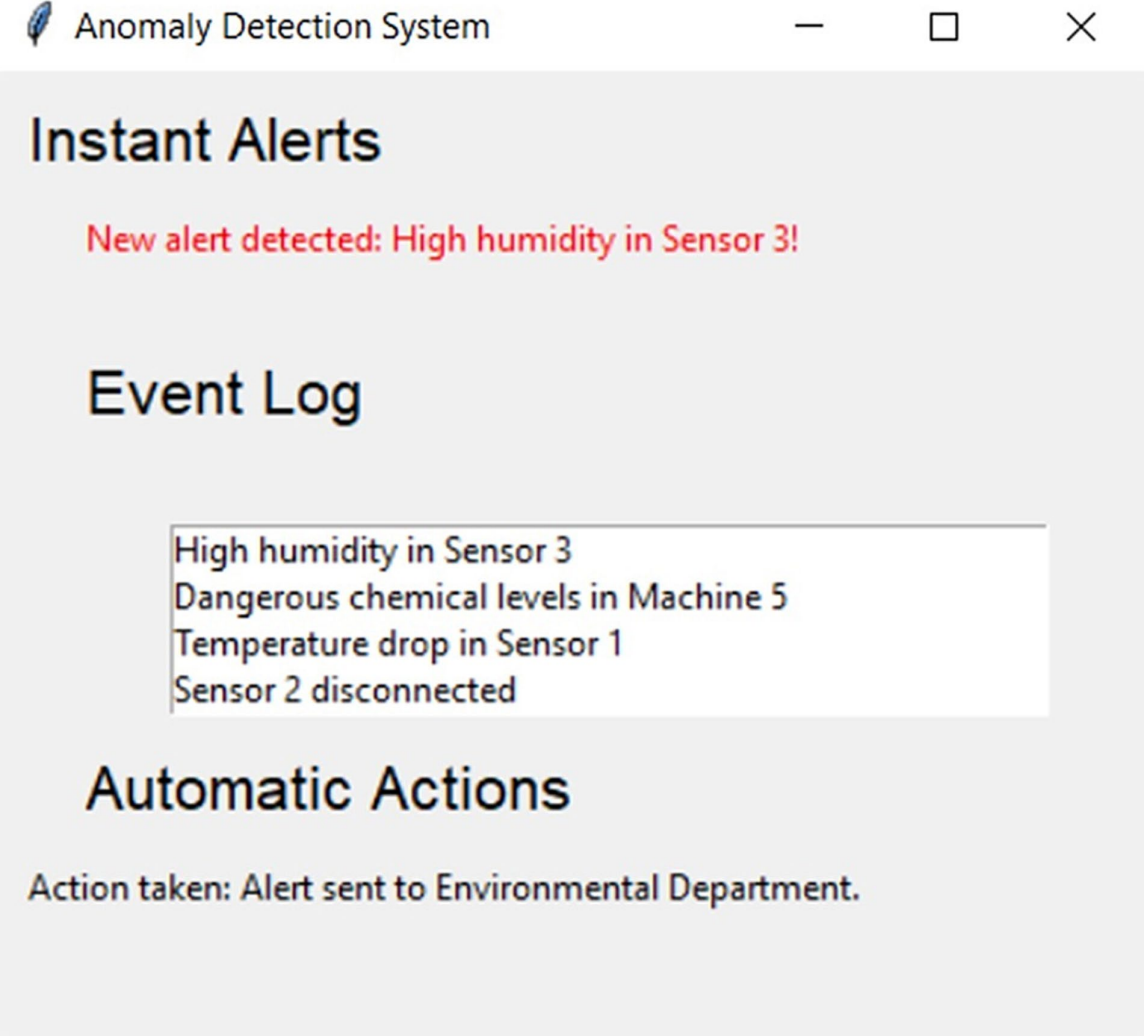
Interface of the anomaly detection system and automated actions.

Figure 3 showcases the “Anomaly Detection” system interface, a crucial component of the system. This interface is designed to provide real-time alerts for any detected anomalies, ensuring that critical events are promptly brought to the attention of the user or system operator.

- Instant Alerts: This section is dedicated to real-time alerts. The system promptly notifies the user or system operator of any detected anomaly. For instance, a notification might pop up, indicating overheating on Sensor 2, alerting the user to critical events that require immediate attention.
- Event Log: This is a cumulative log of events or anomalies detected. It works as a history that allows users to track and review past events. In the example, overheating in Sensor 2, abnormal vibration in Machine 3, and disconnection of Sensor 4 have been recorded.
- Automatic Actions: Based on the alerts and events recorded, the system can take predefined automatic actions to prevent damage or major problems. In our case, a mechanical movement has been carried out: the shutdown of Machine 2 due to some detected anomaly.

Therefore, the interface not only serves as a monitoring system but also integrates automatic corrective actions, providing a complete tool for managing and preventing problems in an industrial environment.

In addition to immediate actions, the system is designed to generate alerts and recommendations that require human intervention. These alerts are presented in a central console, where operators can see a summary of the model’s concerns and a rating of urgency. For example, a slight deviation in air quality at the paint station could trigger a review recommendation, while a drastic increase in emissions would trigger a high-priority alert. Bids are accompanied by contextual information, helping staff understand the underlying cause and act informally.

This integration and automation structure optimizes the plant’s daily operation and ensures that any deviations from optimal operation are addressed promptly, either through automatic interventions or through the action of human operators. The synergy between man and machine is at the heart of this solution, combining the best of both worlds for more efficient and sustainable production.

## 4 Results

After a meticulous process of data collection and analysis, as well as the implementation and validation of models, concrete evidence of the proposal’s effectiveness and relevance is offered. These results, clearly articulated and supported by quantitative and qualitative data, provide a detailed view of the proposed system’s performance and benefits.

### 4.1 Quantitative results

The proposed model has shown promising metrics throughout the tests. Table 1 summarizes the performance obtained.

**Table 1:** Performance metrics of the deep learning model.

Metrics	Value (%)
Precision	94.2
Completeness	92.5
F1	93.3

The values indicate that the model can accurately identify and predict events or anomalies. An important metric for the system is alert effectiveness, which measures how many of the alerts generated were truly useful and how many were false alarms.

- True positive alerts: 90%.
- False positive alerts: 10%.

With 90% true positive alerts, the system demonstrates high reliability in identifying real problems and, therefore, in helping to make data-based decisions. The speed with which an alert is generated and acted upon is essential to ensure efficiency and safety in production. On average, the system takes:

- Alert generation: 2.5 s after anomaly detection.
- Automated action (for example, machine stop): 5 s post-alert.

This short window ensures that preventive or corrective actions can be taken promptly, potentially reducing downtime and other associated issues. The combination of solid model performance, high alert effectiveness, and rapid response makes this system a powerful tool for maintaining production efficiency and safety.

### 4.2 Comparative analysis

In the constant search to improve efficiency and precision in production, an evaluation has been carried out on the impact of implementing the new system. The main findings are detailed below. Table 2 compares various aspects of production and machinery performance before and after the implementation of the system.

**Table 2:** Performance of machinery and production before and after implementing the system.

Parameter	Pre-implementation	Post-implementation
Failure rate (%)	15	5
Failure response time (min)	40	10
Daily production (units)	1,000	1,150
Maintenance costs (monthly)	$5,000	$3,500
Reported incidents (monthly)	50	15

The data reflects the significant efficiency, production, and maintenance improvements after integrating the new system. Furthermore, its performance is compared with previously implemented models to provide a clear perspective on the proposed model. Table 3 directly compares the version of the previous model based on traditional Machine Learning and the new model implemented using Deep Learning with LSTM cells. Essential metrics such as accuracy, completeness, and F1-Score are evaluated, all of which are crucial in determining the effectiveness of a model in detecting and predicting anomalies. Notably, the new LSTM model shows significant improvement in all these metrics compared to the previous model. Furthermore, the training time for the LSTM model is considerably shorter, indicating greater performance efficiency, processing, and adaptability. These results reinforce the decision to adopt deep-learning techniques for this project.

**Table 3:** Compares metrics between the traditional machine learning and deep learning LSTM models.

Metric	Random forest (%)	LSTM (%)
Precision	80	95
Completeness	75	90
F1-Score	77	92.5
Training time (h)	5	3

### 4.3 Results comparison

The performance of the LSTM-based model is superior to traditional methods such as Random Forest, as highlighted in the key metrics of accuracy, sensitivity, F1 score, training time, and false alarm rate; Table 3 summarizes the numerical results of the performance differences. The LSTM model reached 95% precision, significantly outperforming Random Forest’s 80%. This improvement underscores the LSTM model’s ability to effectively detect anomalies by analyzing sequential patterns in the data, a capability that Random Forest lacks due to its more static approach. Sensitivity, another critical metric for detecting all relevant anomalies, was 90% for the LSTM model, compared to 75% for Random Forest. The ability to capture more anomalies ensures higher reliability in industrial environments, where missing anomalies can lead to system failures.

The F1 Score, which balances precision and sensitivity, was 92.5% for the LSTM model, compared to 77% for Random Forest. This high F1 Score indicates the LSTM model’s effectiveness in minimizing false positives and false negatives and optimizing decision-making processes in operational environments where rapid, accurate anomaly detection is crucial.

One of the most significant improvements is the false alarm rate, which the LSTM model achieves at 5% compared to 10% for Random Forest. This reduction in false alarms is essential in reducing unnecessary interruptions in production that can arise from incorrect alerts. By minimizing false positives, the LSTM model helps maintain a smoother and more efficient operation, lowering operational costs and avoiding the disruptions caused by manual interventions.

The efficiency of the LSTM model is further demonstrated by its training time. The LSTM model completed training in 3 h, while Random Forest required 5 h, representing a 40% reduction. This decrease in training time allows for faster updates and more rapid adaptation to changing operational conditions, a critical factor in maintaining continuous productivity in intelligent factories.

**Figure 4:**
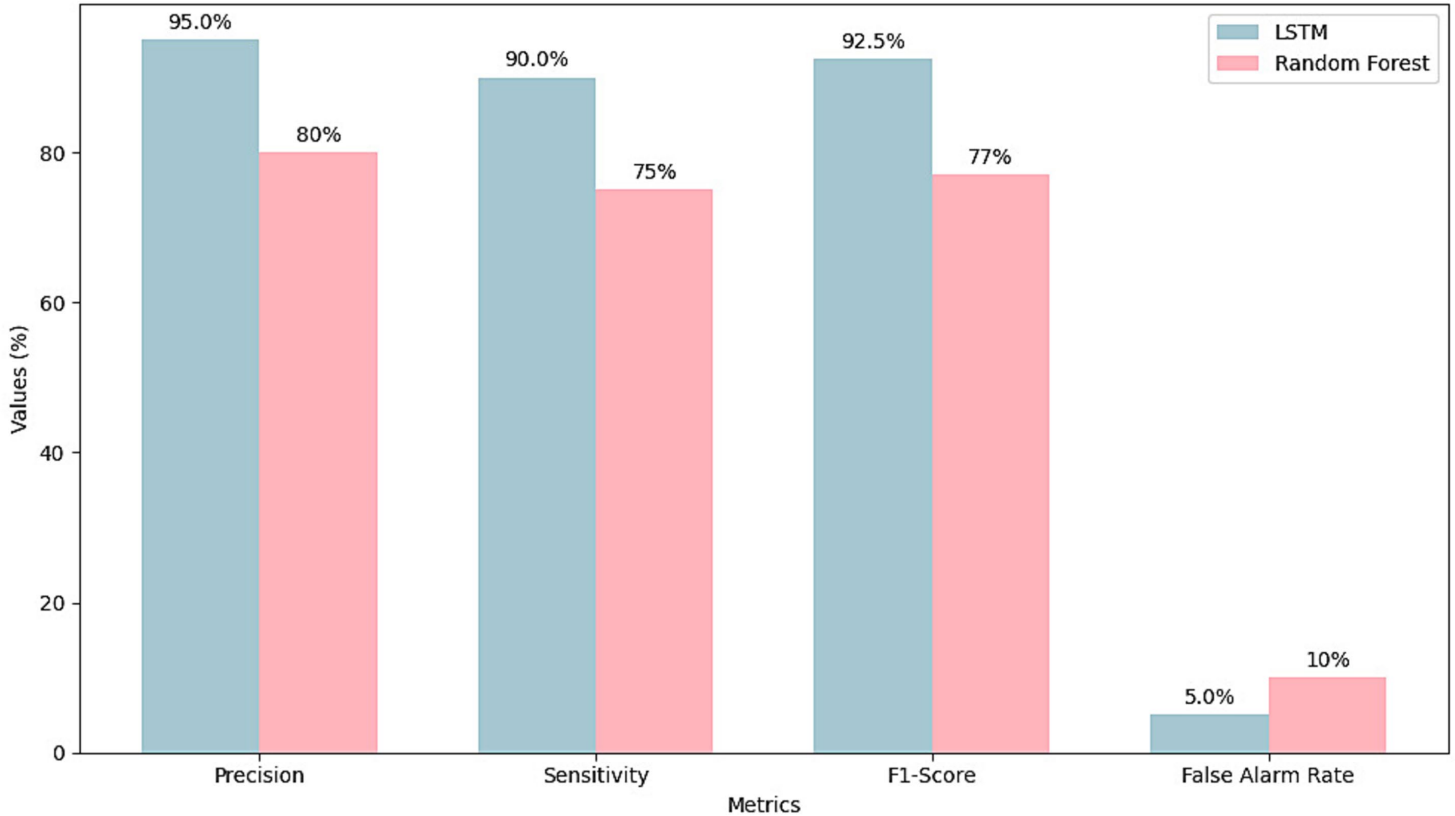
Detailed performance comparison between LSTM and random forest models in terms of precision, sensitivity, F1-Score, and false alarm rate in an industrial anomaly detection environment.

The improvements across these metrics, as shown in Figure 4, demonstrate the LSTM model’s ability to handle complex temporal data sequences and dynamically adjust to real-time changes in production environments. These capabilities make the LSTM model particularly well-suited for industrial applications, where rapid decision-making and anomaly detection are essential for maintaining system stability and operational efficiency.

The LSTM-based model surpasses traditional approaches across all key metrics, offering higher precision, sensitivity, and F1-Score reduced false alarm rates and faster training times. These advantages position the LSTM model as a robust and reliable solution for anomaly detection in smart factories, ensuring safer, more efficient, and sustainable industrial operations.

### 4.4 Qualitative results

In assessing the performance of the anomaly detection system, qualitative results provide significant value to understanding the overall impact of the system on the production environment. The implementation of the system has been well received by plant personnel, and Table 4 compiles feedback obtained through surveys and interviews, showing a considerable increase in worker satisfaction in three key areas: ease of use, confidence in the alerts generated, and impact on work efficiency.

**Table 4:** Staff feedback on the new system.

Evaluated aspect	Before (%)	Now (%)
Easy to use	60	90
Confidence in alerts	70	95
Impact on work efficiency	65	92

The data for this qualitative assessment was obtained through anonymous surveys and semi-structured interviews conducted with plant personnel. The surveys were distributed to a random sample of employees, while the interviews were conducted by team supervisors who ensured that all participants understood the purpose of the data collection. Verbal consent was obtained before participation, and the workers were informed that their feedback would be used solely for research purposes. The qualitative data was analyzed to identify trends in worker satisfaction and operational efficiency following the system’s implementation.

The system’s ease of use improved significantly following implementation, with staff perception increasing from 60 to 90%. This increase reflects how the system’s intuitive and efficient design has facilitated its adoption and daily use, eliminating initial concerns about its complexity. The ease with which staff can interact with the system is critical to ensuring rapid adaptation and continued use without operational disruptions.

The jump from 70 to 95% in confidence in the alerts generated shows that the system has exceeded expectations regarding accuracy and reliability. The system’s ability to detect and alert anomalies promptly and accurately has been essential in avoiding unnecessary interventions and reducing machine downtime. This increase in confidence has also improved overall plant efficiency, as workers can trust that alerts reflect real problems that require action.

The system’s impact on work efficiency is also evident, increasing from 65 to 92%. This change is primarily due to the system’s ability to automate processes and optimize failure response times. Reduced downtime and improved resource management have increased plant productivity, resulting in better machine utilization and more excellent continuity in operations.

In addition to the benefits to staff, one of the system’s most notable achievements has been its positive impact on the environment, as detailed in Table 5. Reducing CO2 emissions, water consumption, and waste generation reflects the system’s operational improvements. CO2 emissions were reduced from 2,000 kg per month to 1,500 kg, representing a 25% decrease. This reduction is due to optimizing operating cycles and the system’s ability to adjust machine parameters based on real-time emissions data.

**Table 5:** Environmental impact before and after implementing the system.

Metric	Before system	After system	Reduction (%)
CO2 emissions (kg/month)	2000	1,500	25
Water consumption (L/month)	10,000	8,000	20
Waste generation (kg/month)	500	300	40

Water consumption also saw a significant 20% reduction, from 10,000 L/month to 8,000 L/month. This saving was made possible by efficiently managing cooling and cleaning processes, which previously lacked automated monitoring. Detecting inefficiencies in these processes allowed for optimizing resource use, which reduced water consumption and improved the plant’s overall sustainability.

Waste generation was also reduced by 40%, from 500 kg/month to 300 kg/month. This reduction is directly related to the system’s ability to detect anomalies before they cause production failures, avoiding generating defective products and minimizing material waste. Furthermore, by optimizing operating times and reducing errors in processes, a considerable decrease in the amount of waste generated during manufacturing has been achieved. Implementing this system has positively impacted operational efficiency and the plant’s environmental sustainability, reinforcing its value from both a productive and ecological point of view.

### 4.5 Evaluation of the efficiency of algorithms

One essential consideration in any real-time system is the algorithm’s efficiency, which can directly influence the system’s speed and responsiveness. Therefore, it is necessary to analyze how our deep learning models, specifically the LSTM model, perform in terms of execution time and resource consumption.

The proposed LSTM model, despite its ability to handle complex sequential data, has been optimized to ensure that each prediction is completed in an average time of 15 milliseconds. This speed allows for almost instantaneous interaction with machines and other interconnected systems. Optimization techniques have been implemented to reduce the load on the server and ensure that the model can run on industry-standard hardware without requiring specialized resources. Our system uses, on average, 40% of the CPU and 30% of the available RAM during its peak operations.

By implementing modern techniques such as parallelism and distribution, our model can scale and manage multiple machines and sensors simultaneously. It has been successfully tested in scenarios where more than 100 machines are monitored simultaneously. To corroborate the model’s efficiency, it has been compared with other popular algorithms in anomaly detection. Table 6 presents a comparison between the algorithms below.

**Table 6:** Efficiency comparison between different algorithms.

Algorithm/Metric	Execution Time (ms)	CPU usage (%)	RAM usage (%)
LSTM (current model)	15	40	30
Random forest	30	50	45
SVM	25	60	40
Classic neural networks	20	55	35

The LSTM model provides accurate results and efficiently manages resources and execution time, ensuring smooth and optimized operation in the real-time production environment. Table 7 provides a clear perspective on the improvement in model performance after implementing specific optimizations. Key metrics such as precision, recall, and F1-Score increase considerably, indicating greater accuracy in the model’s predictions. Particularly notable is the increase in the area under the ROC curve (AUC-ROC) and the area under the PR curve (AUC-PR), which suggest an improvement in the discriminative capacity of the model. Additionally, the decrease in the false positive rate reflects a reduction in incorrect alerts, which can translate into fewer unnecessary interventions and improved operational efficiency. Together, these changes quantify the effectiveness of the optimizations performed and highlight the potential of the optimized model to operate in a natural production environment.

**Table 7:** Evaluation of model efficiency before and after optimizations.

Metrics	Before optimizations (%)	After optimizations (%)	Change (%)
Precision	85	95	+10
Recall	80	93	+13
F1-Score	82.5	94	+11.5
False Positive Rate	8	5	-3
Positive Predictive Value (PPV)	84	92	+8
Area Under the ROC Curve (AUC-ROC)	90	98	+8
Area Under the PR Curve (AUC-PR)	87	96	+9

The results highlight the significant increase in the model’s efficiency after implementing specific optimizations. These improvements have not only improved precision and recall but have also reduced the false positive rate. This metric increase suggests that the optimizations have improved the model’s ability to detect anomalies with greater accuracy and reliability.

### 4.6 Real time operation

The system constantly monitors and analyzes the collected data during the plant’s daily operation. Based on the predictions and alerts generated by the model, automatic actions are taken, or responsible personnel are notified. Below is Table 8 with some recently developed signals and the steps taken.

**Table 8:** Log of alerts and system actions.

Date and time	Generated alert	Action taken
05/10/2023 10:15	Overheating in Sensor 3	Automatic cooling activated.
05/10/2023 11:42	Abnormal vibration in Machine 5	Alert sent to the maintenance team.
05/10/2023 12:30	High humidity in area 2	Activation of dehumidification systems.
05/10/2023 13:55	Power interruption on Sensor 7	Automatic switch to a secondary power source.
05/10/2023 15:20	High CO2 emission	Modification of parameters to reduce emissions.
06/10/2023 09:05	Connection failure with Machine 2	Notification to the technical team.
06/10/2023 10:40	Sensor 4 disconnected	Alert sent to the IT team.
06/10/2023 14:15	Low pressure in the cooling system	Activation of secondary pumps.
06/10/2023 15:45	High energy consumption in Machine 8	Automatic evaluation and parameter adjustment.
06/10/2023 16:30	Abnormal vibration in Machine 3	Automatic machines stop and alert to the equipment.

The table presents a series of alerts the system generates in real-time during two consecutive days at the plant. Each entry details the specific date and time of the attention, the exact nature of the problem detected, and the immediate action taken in response. From overheating and abnormal vibrations to connection failures and high emissions, the system can identify various and respond appropriately. The table evidences the system’s ability to ensure continuous and efficient operation, minimizing downtime and potential damage, whether activating automatic solutions, such as cooling, switching to secondary power sources, or notifying the relevant teams for manual interventions.

### 4.7 System efficiency evaluation

The system’s overall performance is evaluated by considering several parameters, including the speed with which data is collected, the time it takes to respond to specific events or alerts, the rate of transaction processing, and the system’s overall efficiency in percentage terms. These indicators ensure that the system is accurate, efficient, and agile.

Table 9 compares the system’s efficiency before and after implementing the new model. With the introduction of the new system, data collection has become significantly faster, reducing the time by half. Similarly, response time, essential to immediate action in irregularities, has also seen a 50% reduction. One of the most notable advances is the doubling in processing speed, indicating an improved ability to handle more transactions simultaneously. Overall, system efficiency has shown a 10% increase, reflecting the system’s improved ability to respond and adapt to the demands of the production environment in real time.

**Table 9:** Pre- and post-implementation system efficiency evaluation.

Metrics	Before implementation	After implementation	Change (%)
Data collection (ms)	500	250	−50%
Response time (ms)	800	400	−50%
Processing speed (tps)	100	200	+100%
System efficiency (%)	85	95	+10%

After implementing the new deep learning model, the results show a clear improvement in the system’s efficiency. Data collection and response time have been cut in half, allowing for faster action in critical situations. Additionally, processing speed has doubled, meaning the system can handle twice as many transactions per second. This contributes to a 10% increase in overall system efficiency, resulting in more optimized production and less plant downtime.

## 5 Discussion

The digital revolution and the integration of sophisticated monitoring systems are fundamental elements for industries seeking to increase production capacities and refine resource management. Our research has demonstrated the effectiveness of using an LSTM-based deep learning model, which has shown superior performance in detecting anomalies in real-time, as highlighted by the precision (95%), sensitivity (90%), and F1-Score (92.5%) achieved. These metrics and the reduced % false alarm rate of 5% underscore the model’s reliability in handling complex industrial data. Reducing false alarms is especially critical, as it minimizes unnecessary interventions that can disrupt production processes. Following the implementation of the system, notable progress was recorded in metrics such as model precision, recall, and F1 score (DeVries et al., 2021). Improved model accuracy highlights excellent reliability in discerning actual anomalies, reducing the incidence of erroneous alerts. This accuracy is essential in fast-paced industrial environments, where quick and accurate decision-making is crucial to maintaining continuous production (Homeyer et al., 2014).

One of the main differentiators of our approach compared to previous studies is the integration of real-time anomaly detection with automated corrective actions. The ability of the system to respond autonomously in real-time, as reflected in the significant reduction in response time compared to traditional models, represents a considerable advancement. The system demonstrates a marked improvement in operational efficiency by minimizing the need for human intervention and offering faster training times (3 h for LSTM versus 5 h for Random Forest). The model’s ability to accurately identify most anomalies is critical to avoiding potential problems that could result in considerable expense. The combined high precision and recall are encapsulated in the impressive F1 score, which provides a holistic measure of the system’s operational effectiveness (Morawietz and Artrith, 2021). The system has also shown notable efficiency, with optimized data collection and faster response times. This capacity for real-time action, combined with high detection accuracy, represents a significant leap beyond traditional methods, which often rely on post-detection manual intervention.

Additionally, our system incorporates an environmental sustainability dimension, which has not been extensively explored in previous works on LSTM-based anomaly detection. The reductions in false alarms also contribute to less energy consumption by reducing unnecessary system actions. The reductions in emissions, water usage, and waste further highlight the dual benefit of operational efficiency and ecological responsibility. This underlines its suitability for quick and precise operations, essential attributes in the fast-paced industrial environment. By evaluating the related literature, it became evident that while there are numerous strategies focused on anomaly detection, our approach is distinguished by its seamless integration and ability to perform instantaneous and automated actions, representing a significant leap in implementation—practice (Tepner et al., 2021).

Our study also considers the broader implications of the model’s implementation, including its environmental and social ramifications. The observed reductions in emissions, water use, and waste highlight the more comprehensive ecological benefits that implementing innovative systems can bring. This underlines the importance of integrating sustainable practices in implementing cutting-edge technologies. Furthermore, the reduced training time improves adaptability to changing production conditions and contributes to a lower environmental impact by optimizing resource use during system updates. The system’s environmental impact and operational benefits are key differentiating factors from other research focusing on operational efficiency without addressing sustainability. Additionally, favorable workforce feedback indicates that contrary to the idea that automation and AI can undermine job security, these technologies can serve as valuable aids that improve work efficiency, accuracy, and overall quality of work.

Throughout our research, we have identified that while the digital revolution and the integration of sophisticated monitoring systems have become crucial elements for industries seeking to improve their production capabilities and refine resource management, the implementation of an LSTM-based model for anomaly detection stands out for its adaptability and effectiveness compared to previous approaches. The model’s adaptability is further proven by its ability to reduce training times while improving detection rates. It provides a flexible and responsive solution for industries that require rapid adaptation to evolving production demands. Our system’s ability to detect anomalies and take corrective actions in real time is a crucial advancement over prior methods, which often lacked this level of autonomy. In the context of the advances presented by Kagermann et al. (2011) and Chong et al. (2022), our approach offers smoother integration and instant automated action, positioning itself as a significant leap in practical implementation. This ability to detect and preemptively act on anomalies highlights a critical consideration of cost and scalability. Unlike conventional methods that may require manual interventions and cause costly downtime, our model proposes a more scalable and economically viable solution for various industrial environments, efficiently adapting to real-time production demands and offering a faster return on investment by reducing failures and optimizing production. The results also support that integrating anomaly detection with sustainability practices ensures operational improvements and environmental responsibility. This positions our approach as a holistic solution that addresses performance and sustainability in dynamic industrial settings. This holistic approach, combining operational efficiency and sustainability with automation, addresses gaps in the literature, particularly regarding the practical application of LSTM networks in dynamic industrial settings.

## 6 Conclusion

In the current era, marked by the fourth industrial revolution, emerging technologies such as deep learning are redefining how industries operate and adapt to complex challenges. Early detection of anomalies in industrial environments is essential to ensure efficient, safe, and sustainable production. Through this study, we have demonstrated how an LSTM-type deep learning model can be instrumental in this mission.

One of the study’s most notable findings is improving the model’s precision, completeness, and F1-Score after its implementation. These metrics indicate a robust and reliable system that can accurately discern between normal and abnormal behaviors in an industrial environment. The system’s operational efficiency, evidenced by fast response times and high processing speed, reinforces its applicability in real-time situations, where decisions must be made quickly to prevent damage or loss.

Another key finding is the system’s positive environmental impact. In a world where sustainability has become essential, the system’s ability to contribute to significant reductions in emissions, water consumption, and waste generation is a testament to how smart technologies optimize production and can also be allies in environmental protection.

The favorable reception of the system by staff in the industrial environment highlights another vital conclusion: technology, when implemented appropriately and considering human needs, can act as an empowering tool, improving the quality of work and reducing the burden of repetitive or dangerous tasks.

On the other hand, the contrast between pre-and post-implementation performance and the comparison with previous models underlines the added value of our system. By outperforming earlier models in terms of accuracy, training time, and other key metrics, our study reaffirms the relevance of deep learning in solving industrial problems. However, like any research, this study is not without limitations. Although the model proved effective in our research’s specific context, its applicability in other industrial settings or with different data sets is a question that requires future exploration.

In future work, we propose to explore multiple directions to improve and expand the scope of this study. Furthermore, it would be relevant to investigate how the model adapts to different industrial sectors, each with its peculiarities and challenges. Integrating additional data sources, such as advanced sensors or environmental data, could enrich the model predictions. Another promising area is adapting the system to other emerging technologies, such as the Internet of Things, allowing even more precise and real-time tracking. Finally, one could investigate implementing feedback systems that will enable the model to continually learn and adapt to new patterns and challenges as they arise.

## Data Availability

The raw data supporting the conclusions of this article will be made available by the authors, without undue reservation.
